# Methacrylate peak determination and selection recommendations using ATR-FTIR to investigate polymerisation of dental methacrylate mixtures

**DOI:** 10.1371/journal.pone.0252999

**Published:** 2021-06-09

**Authors:** António H. S. Delgado, Anne M. Young

**Affiliations:** 1 Division of Biomaterials and Tissue Engineering, UCL Eastman Dental Institute, London, United Kingdom; 2 Centro de Investigação Interdisciplinar Egas Moniz (CiiEM), Instituto Universitário Egas Moniz, Monte de Caparica, Portugal; Indian Institute of Technology Kharagpur, INDIA

## Abstract

Investigation of polymerisation kinetics using ATR-FTIR systems is common in many dental studies. However, peak selection methods to calculate monomer-polymer conversion can vary, consequently affecting final results. Thus, the aim of this study is to experimentally confirm which method is less prone to systematic errors. Three commercial restorative materials were tested–Vertise Flow (VF), Constic and Activa Bioactive Restorative Kids. Firstly, Attenuated Total Reflectance Fourier Transform Infra-Red (ATR-FTIR) (Spectrum One, Perkin-Elmer, UK) spectra of monomers were acquired—10-methacryloyloxy decyl dihydrogen phosphate (10-MDP), bisphenol-A glycidyl dimethacrylate (Bis-GMA), 2-hydroxyethyl methacrylate (HEMA), triethyelene glycol dimethacrylate (TEGDMA) and urethane dimethacrylate (UDMA) to investigate proportionality of methacrylate peak heights versus concentration. Spectral changes upon light exposure of 2 mm discs of the restorative materials (irradiated for 20 s, LED curing unit 1100–1330 mW/cm^2^) were assessed to study polymerisation kinetics (*n* = 3), with continuous acquisition of spectra, before, during and after light exposure. Peak differences and degrees of conversion (D_C_ %) were calculated using 1320/1336, 1320/1350 and 1636/1648 cm^-1^ as reaction/reference peaks. Inferential statistics included a MANOVA and within-subjects repeated measures ANOVA design (5% significance level). Proportionality of methacrylate peak height to concentration was confirmed, with the 1320/1352 cm^-1^ peak combination showing the lowest coefficient of variation (8%). Difference spectra of the polymerisation reaction showed noise interference around the 1500–1800 cm^-1^ region. Across the different materials, D_C_ % results are highly dependent upon peak selection (*p*<0.001), with higher variability associated to the 1636 cm^-1^. Significant differences in the materials were only detected when the 1320 cm^-1^ peak was used (*p*<0.05). Within the same materials, methods were significantly different for Constic and Activa (*p*<0.05). It is possible to conclude that the 1320 cm^-1^ peak is more adequate to assess polymerisation of methacrylates and is therefore recommended.

## Introduction

Fourier Transform Infra-Red Spectroscopy (FTIR) is able to quantify infra-red light absorbance and transmittance, resulting from changes in the dipole moment of bonds in molecules, which display vibrational patterns [1]. It is a technique widely used for material characterization, namely organic materials such as polymers, with high applicability in many different fields [2, 3]. In dentistry specifically, it is understood as a reference technique for the study of polymerisation kinetics of dental materials, especially useful when coupled with Attenuated Total Reflectance (ATR) accessories, as recognized by the Academy of Dental Materials [4].

Resin composites, a universally used restorative material in dentistry, are made of an organic resin phase of methacrylate derivatives (such as Bisphenol-A glycidyl dimethacrylate–Bis-GMA, triethylene glycol dimethacrylate–TEGDMA or urethane dimethacrylate–UDMA), combined with a filler phase of glass particles and a photoinitiator system [5]. Typically, free-radical addition polymerisation reactions in these materials are activated by blue light, and they can be monitored using real-time ATR-FTIR systems [6]. Over the past years, several studies across the dental literature have successfully measured the degree of conversion (D_C_ %) of methacrylate monomer bond changes, using such technique [7–10]. Methacrylate polymerisation monitoring is researched not only with resin composites but also with other dental materials containing resin monomers, such as dental adhesive systems, resin-modified glass ionomers and compomers [11–14].

To calculate this parameter, in most studies, the ratio between the reactionary methacrylate 1640 cm^-1^ [*v*(C = C)] and the 1610 [*v*(C = C)] cm^-1^ peak as the internal standard reference, are used in Bis-GMA mixtures. However, as O-H bending vibrations absorb in the same region, and spectral contributions of carbonyl groups may affect the 1640 cm^-1^ peak, alternative peak selections are recommended [15]. The 1320 cm^-1^ [*v*(C-O)] has been successfully employed for the study of polymerisation in dental composites and has been preferred over the 1640 cm^-1^ peak in a number of cases [9, 11, 16]. Despite having some studies acknowledge the variability regarding peak selection, the 1320 cm^-1^ peak is still underappreciated [17]. Repeatability of the ATR-FTIR technique also demands investigation, as it is important in confirming its ability to detect small changes within sample repetitions or between different time points, in scenarios such as reactions or material aging [18, 19].

To measure D_C_ % in light-curable materials, other direct measuring techniques have been used in past studies, such as Raman spectroscopy [15]. Raman shows comparable results to FTIR in resin composites [20], but it is seldom used to acquire continuous spectra which are useful for the study of the evolution of the polymerisation reaction and its kinetics. It may also be time consuming, due to Raman scattering being a weak effect, with signal strength issues, leading to a steeper learning curve [21]. ATR-FTIR sample preparation and method is a simple, rapid and easy access tool that allows spectra to be acquired before, during and after light curing, allowing the operator to cure *in situ* without interfering with the measurement [13]. ATR-FTIR has also been proven convenient to study setting kinetics in glass ionomer cements, calcium silicates and bone cements [11, 12, 22, 23].

The aim is then to assess whether final D_C_ (%) is dependent upon peak selection method, by investigating which peak choices are less prone to systematic errors, shedding light on which method should be used to determine D_C_ (%) in polymerisation of methacrylates.

## Materials and methods

### Raw spectral acquisition

Individual monomers 10-methacryloyloxydecyl dihydrogen phosphate (10-MDP, DMHealthcare, San Diego, CA, USA, code P01030), 2-hydroxyethyl methacrylate HEMA (DMG, Hamburg, Germany code 11220), Bis-GMA (Polysciences, Warrington, PA, USA, code 03344), UDMA (DMG, Hamburg, Germany, code 100112) and TEGDMA (DMG, Hamburg, Germany, code 100102) were purchased for FTIR spectral acquisition, using a diamond ATR accessory (Golden Gate ATR, Specac Ltd., Orpington, UK) attached to a FTIR spectrometer (Spectrum One, Perkin-Elmer, MA, USA). All spectra were acquired from 700–4000 cm^-1^ at a resolution of 4 cm^-1^. Spectrum TimeBase v. 3.1.4 (Perkin-Elmer, MA, USA) was used for data processing.

ChembioDraw Pro v.19.1 (Perkin-Elmer, MA, USA was used to draw the chemical structure of each monomer for peak assignments and interpretation.

In order to confirm methacrylate peak heights are proportional to the concentration of methacrylate groups in each monomer, an equation relating to the moles of monomer per unit volume was used,

$$\frac{P_{r}-P_{b}}{\left(\frac{N_{meth}\times \rho }{M_{w}}\right)}$$ (1)

where *P_r_* represents the peak height of the reaction peak, and *P_b_* represents the peak height of the baseline peak, in absorbance units. *N_meth_* represents the number of methacrylate groups in the monomer, *ρ* represents the density (g/cc), while *M_w_* represents the molecular weight (g/mol).

### Polymerisation kinetics using ATR-FTIR

Three different restorative materials were tested in this study. Two metal circlips (1 mm thickness x 10 mm internal diameter) were used to contain resin composite samples of 2 mm thickness, total, of each material (*n* = 3)–Vertise Flow (VF) (Kerr/KaVo, Orange, Ca, USA), Constic (DMG, Hanau, Germany) and Activa Bioactive Restorative Kids (Pulpdent, Watertown, MA, USA) (Table 1). Each material was dispensed into the circlips, which were on an ATR (Specac Ltd., UK) diamond crystal plate. An acetate sheet was placed on top of the circlips, and a glass slide was used to apply pressure to the material. The top surface of the material was irradiated with a single emission peak light emitting diode (LED) light curing unit (LCU) (Demi Plus, Kerr, Orange, CA, USA) with a power output between 1100 mW/cm^2^–1330 mW/cm^2^, and spectral emission ranging from 450 to 470 nm. FTIR spectra were obtained before, during and after 20 s of light exposure, for a total time period of 1200 s, originating an average of 193 spectra for each repetition. These were acquired over a wavenumber range of 700 to 4000 cm^-1^ at a resolution of 4 cm^-1^, at 37°C. The light curing began 20 ± 5 s after placement of the material and the start of the spectral acquisition.

**Table 1:** Material composition for commercial restorative materials used in this study.

*MATERIAL*	*TYPE*	*COMPOSITION*	
*Vertise™ Flow (Kerr, USA) **VF***	Flowable resin composite	5–10% HEMA, N/A% Bis-GMA, 5–10% UDMA and 1–5% GPDM	Ytterbium fluoride and barium aluminosilicate (66 wt%)
*Constic (DMG, GER)*	Flowable resin composite	15–35% Bis-GMA, <45% TEGDMA and N/A% 10-MDP	Barium aluminosilicate
*Activa™ Kids (Pulpdent, USA)*	Resin modified glass ionomer	44.6% blend of UDMA and other methacrylates with modified polyacrylic acid	Sodium fluoride and silica (56 wt%)

10-MDP: 10-methacryloyloxy decyl dihydrogen phosphate; Bis-GMA: Bisphenol-A glycidyl dimethacrylate; GPDM: glycerophosphate dimethacrylate; HEMA: 2-hydroxyethyl methacrylate; TEGDMA: triethyelene glycol dimethacrylate; UDMA: urethane dimethacrylate.

To calculate the D_C_ (%), the following equation was used (2), where (h_0_) and (h_t_) represent the height of a reactionary methacrylate peak above baseline (reference peak), initially and at time, t, after start of polymerisation respectively.


$$D_{C}=\frac{100(h_{0}-h_{t})}{h_{0}}$$ (2)

For this study, different reaction peaks and bases were selected, to test their effect on determining conversions, and to look at the variability of the data.

- 1320 cm^-1^ [v(C-O)] with 1336 cm^-1^ as baseline
- 1320 cm^-1^ [v(C-O)] with 1350 cm^-1^ as baseline
- 1636 cm^-1^ [v(C = C)] with 1650 cm^-1^ as baseline

A continuous spectral acquisition during polymerisation, without disconnection from the ATR diamond, allows for the continuous monitoring of the exact same material volume during polymerisation. Normalisation by a reference peak is thus not needed.

To investigate spectral changes between the initial and the final time point, while spectra were continuously being acquired, the difference between the final and the initial spectra were taken and studied for the repetitions of the three different materials. To collect and analyse the resulting spectra, a spectral treatment software was used—Spectrum TimeBase (v.3.1.4, Perkin-Elmer, MA, USA). This allowed calculation of the ratio of intensity, on the ATR diamond, obtained with versus without the sample and posterior conversion. Of the data to absorbance versus wavenumber (cm^-1^).

The reaction extent was calculated using the following Eq (3), for the 1320 cm^-1^ reaction peak, without baseline subtraction

$$\zeta =\frac{\left(A_{i}-A_{t}\right)}{{(A}_{i}-A_{f})}$$ (3)

where A is the absorbance of the 1320 cm^-1^ peak without baseline subtraction; *i*, *t* and *f* indicate initial, at time t and final absorbance (determined by extrapolation of absorbance versus inverse time to zero), respectively.

### Statistical analysis

To ensure sufficient sample size, an a priori test of the difference of means of K independent groups, using a one-way ANOVA design, was conducted with statistical software G*Power v.3.1.9.6 for Mac (HHU Düsseldorf, Germany). An alpha of **0.05** was used, with a total sample size of **9** (*n* = 3 for 3 groups), yielding >95% power to detect differences. To assess peak changes, across the repetitions of each sample, mean band maxima of the peaks described above, and standard deviations were calculated for the initial and final spectra. Statistical software package Statistical Package for the Social Sciences—SPSS v. 26.0 (IBM Corporation, Armonk, NY, USA) was used for hypothesis testing. A multivariate analysis of variance (MANOVA) design was employed to compare final D_C_ (%) between different materials, across three independent peak determination methods, while a within-subjects design repeated measures ANOVA was used to compare different peak determination methods within the same materials. Games-Howell post-hoc test was used for the MANOVA, while a Bonferroni multiple comparison post-hoc was used for the repeated measures ANOVA. Assumptions were checked prior to both analyses of variance designs. All inferential analyses were conducted using a significance level of 5%.

## Results

**Fig 1:**
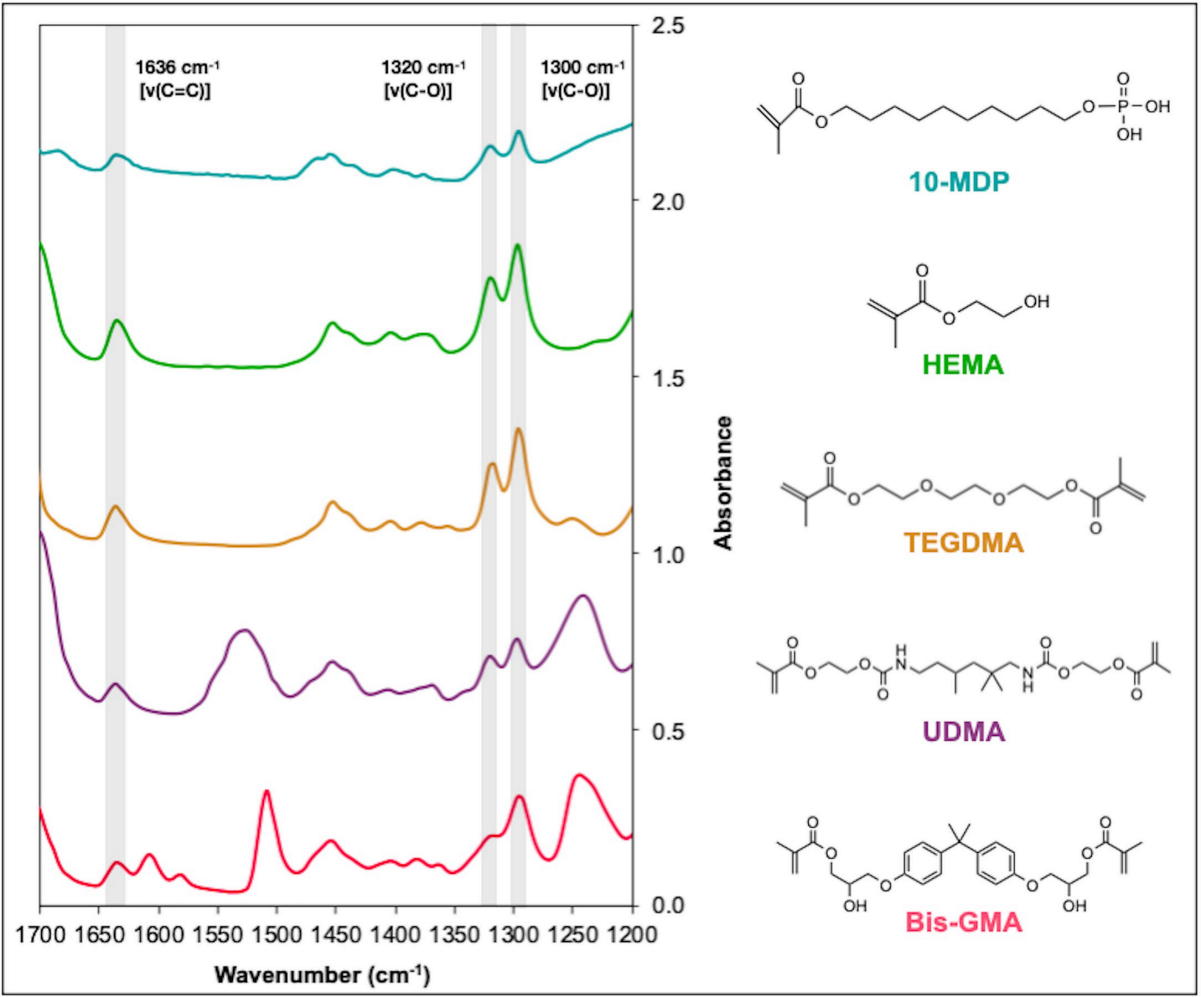
FTIR spectra and chemical structure of dimethacrylates Bis-GMA, UDMA, TEGDMA and monomethacrylates HEMA and 10-MDP. C-O stretch doublet can be seen at 1300 and 1320 cm^-1^ together with 1636 [v(C = C)] bond.

The chemical structure and corresponding FTIR spectra of the individual monomers, with methacrylate peak assignments, used in the chemical composition of the materials that were tested (Table 1) are given in Fig 1.

Assigned methacrylate peak heights for the individual monomers and respective bases are given in Table 2. The 1320 cm^-1^ [v(C-O)] peak, when used as the reaction peak, with the appropriate base, gives results with lower coefficient of variation (CV%), in estimating proportional level of monomer concentration, when compared to the 1636 cm^-1^ peak, showing enhanced repeatability.

**Table 2:** Peak absorbance of individual monomers (Part I), from the spectra acquired and peak height per mole per cc, derived using Eq (1) (Part II), for the different reaction peaks and peak bases.

**Part I**	**Absorbance**					
**Peak wavenumber (cm^-1^)**	10-MDP	HEMA	TEGDMA	UDMA	Bis-GMA	
**Reaction 1320**	0.15	0.28	0.25	0.21	0.20	
**Baseline 1336**	0.08	0.10	0.07	0.11	0.14	
**Baseline 1352**	0.06	0.07	0.07	0.09	0.10	
**Reaction 1636**	0.13	0.16	0.13	0.13	0.13	
**Baseline 1648**	0.09	0.06	0.05	0.08	0.07	
**Part II**	**10-MDP**	**HEMA**	**TEGDMA**	**UDMA**	**Bis-GMA**	
**Peak/base employed**	Peak height derived with Eq 1 (mole^-1^ cc^-1^)					Mean (SD) [CV%]
**1320–1336**	21	21	23	20	14	20 (3.5) [18%]
**1320–1352**	27	25	23	25	23	25 (2.0) [8%]
**1636–1648**	12	14	10	10	13	12 (1.3) [11%]

**Fig 2:**
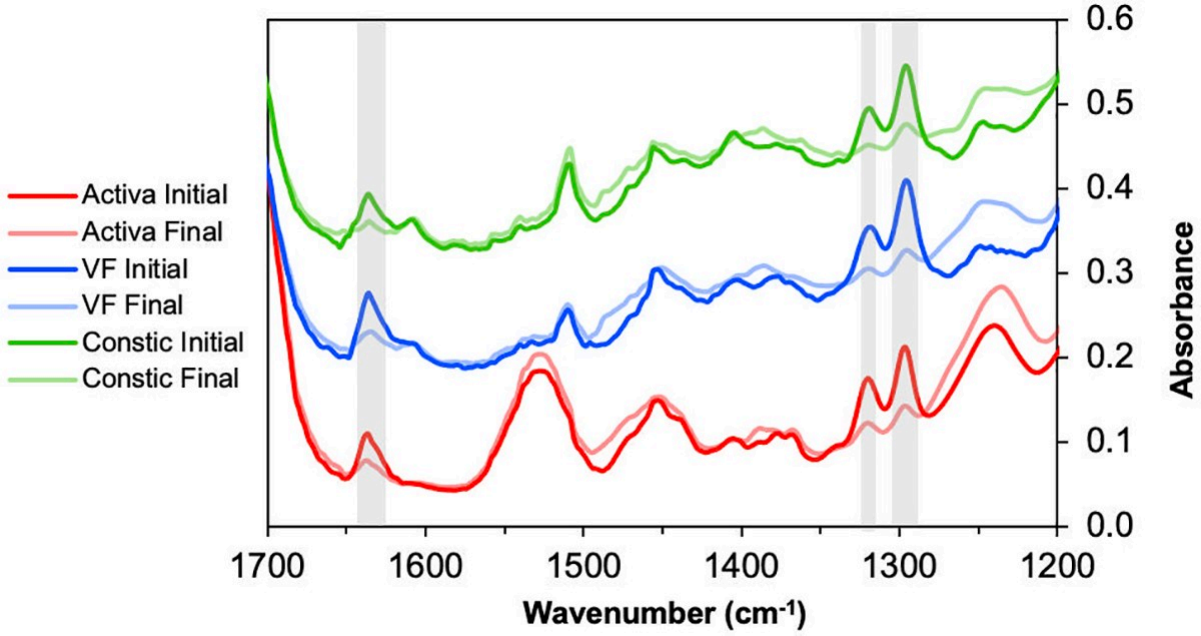
FTIR spectra of VF, Constic and Activa, initial and final (1200 s) spectra. Absorbance changes due to polymerisation can be seen at the C-O stretch doublet at 1300 and 1320 cm^-1^ together with 1636 [v(C = C)] bond, highlighted in grey. Samples with 2 mm thickness, light cured for 20 s (*n* = 3).

FTIR spectra of each restorative material, before and after photopolymerisation, are shown in Fig 2. Spectra of VF and Constic are compatible with the presence of Bis-GMA as a main monomer, with a distinct 1610 aromatic [*v*(C = C)] peak, while Activa shows a dominant UDMA spectra with distinct contributions at 1535 cm^-1^ [δ(N-H)] and 1240 cm^-1^.

**Fig 3:**
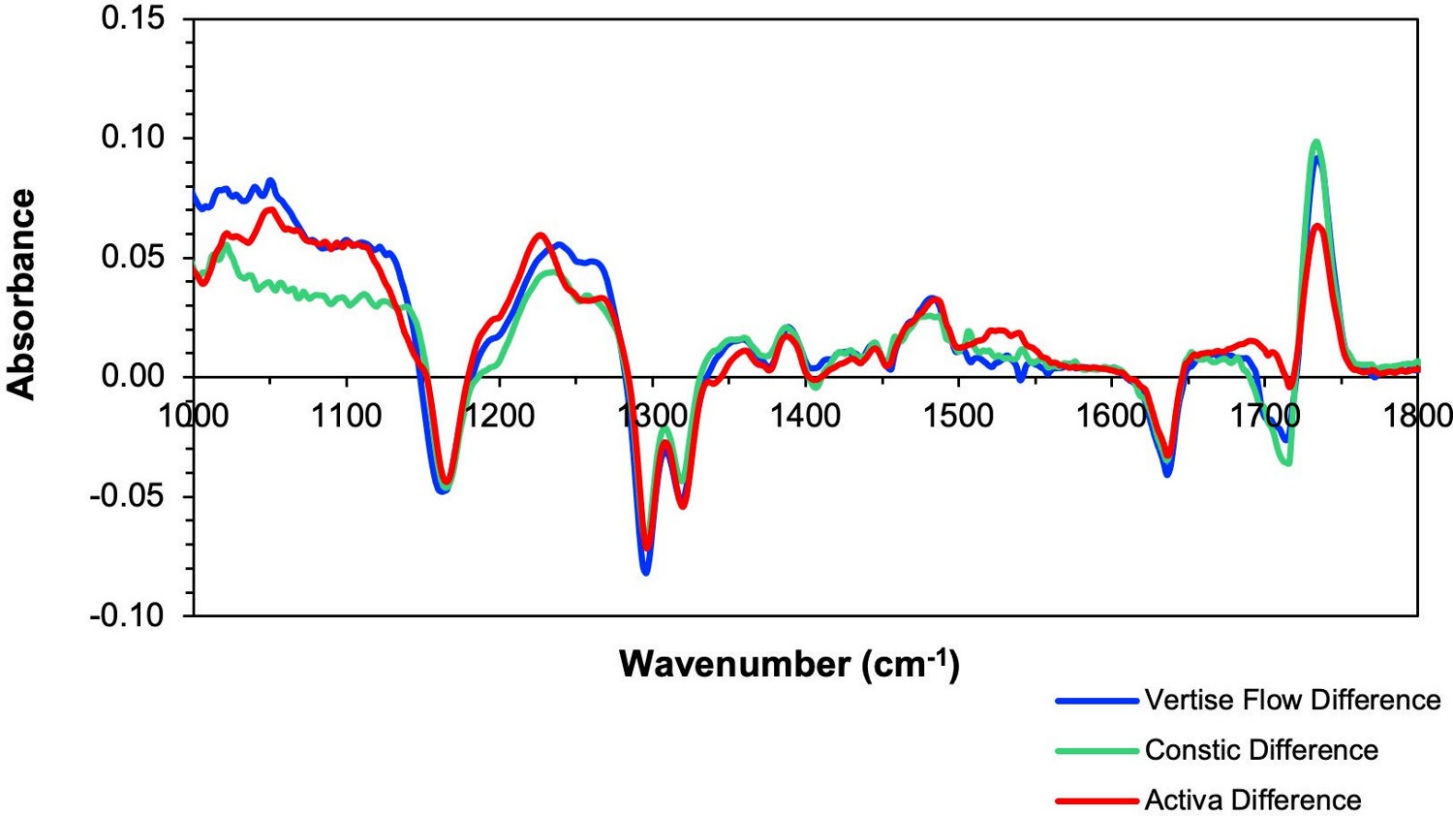
FTIR difference spectra (initial–final spectrum). Graph shows peak shifts owing to the polymerisation reaction. Absorbance changes are seen in the 1160 cm^-1^ [v(C-O-C)], 1300–1320 [v(C-O)] which shift to lower wavenumbers, at 1230 and 1268 cm^-1^, and absorbance change of [v(C = C)] at 1640 cm^-1^. Spectra also show noise around the 1500–1800 cm^-1^ region, with a smooth region between 1100–1500 cm^-1^.

From Fig 2, it is also possible to confirm that the absorbance changes seen in the final spectra are very similar between the three materials, with shifts in the same regions, showing that major changes are due to methacrylate polymerisation. Taking the difference spectra between the initial and final spectrum revealed again changes associated with polymerisation, shown with all the materials in Fig 3. Additionally, the presence of noise in the 1000–1100 and 1500–1800 cm^-1^ regions due to high glass filler content and background water vapour absorbance, respectively, could be detected in some samples. However, no noise was observed between these wavenumber regions.

Peak height differences, respective errors and D_C_ (%) for each material and method are given in Table 3. Final D_C_ (%) means and standard deviations are dependent upon the method chosen, with a statistically significant difference among the groups on a linear combination of the three dependent variables (MANOVA, *p*<0.001, η^2^>0.99). Material differences in respect to conversion are seen with the first two methods, whereas with the last method, conversions are not significantly different (Games-Howell post-hoc, *p*>0.05). With the 1320–1336 cm^-1^ method, VF is significantly different to Constic (*p* = 0.008) and to Activa (*p* = 0.002), while Constic was also significantly different to Activa (*p* = 0.001). Considering the 1320–1350 cm^-1^ method, VF was significantly different to Constic (*p* = 0.035) and Activa (*p* = 0.015). Constic was also significantly different to Activa (*p*<0.001). D_C_ (%) results show higher variability when the 1636 cm^-1^ peak is chosen, with higher standard deviations and coefficients of variation, compared to selecting the 1320–1336 cm^-1^ peaks, where standard deviations do not go above 1 (Table 3).

**Table 3:** Peak heights and resulting D_C_ (%) at 1200 s, for the different peak selection methods.

	VF		Constic		ACTIVA	
	Peak difference	D_C_ (%)	Peak difference	D_C_ (%)	Peak difference	D_C_ (%)
**1320–1336**	0.068 (0.006)	74 (1) [1.8] ^**aA**^	0.062 (0.001)	82 (0) [0.54] ^**aB**^	0.078 (0.001)	64 (1) [2.1] ^**aC**^
**1320–1350**	0.085 (0.004)	76 (3) [4.0] ^**aA**^	0.066 (0.002)	88 (0) [0.65] ^**bB**^	0.094 (0.001)	62 (1) [1.8] ^**bC**^
**1636–1648**	0.075 (0.013)	73 (5) [6.9] ^**aA**^	0.050 (0.005)	76 (3) [3.93] ^**aA**^	0.053 (0.003)	71 (5) [7.3] ^**abA**^

Means and standard deviations (SD) of peak heights, in absorbance units, between repetitions (*n* = 3) and final D_C_ (%) means, (SD) and [CV%] for each method, showing differences. Different lower-case letters indicate significant differences within the same column (Bonferroni, *p*<0.05), while different capital letters indicate differences in the same rows (Games-Howell, *p*>0.05).

Differences in the overall conversion within-group, for different methods are seen in Constic and ACTIVA (Bonferroni post-hoc, *p*<0.05). Within Constic, comparing the 1320–1336 to the 1320–1350 cm^-1^, differences were found (*p* = 0.11), but also between the 1320–1350 and the 1636–1648 cm^-1^ (*p* = 0.044). With Activa, differences were found between the 1320–1346 and the 1320–1350 cm^-1^ (*p* = 0.006).

**Fig 4:**
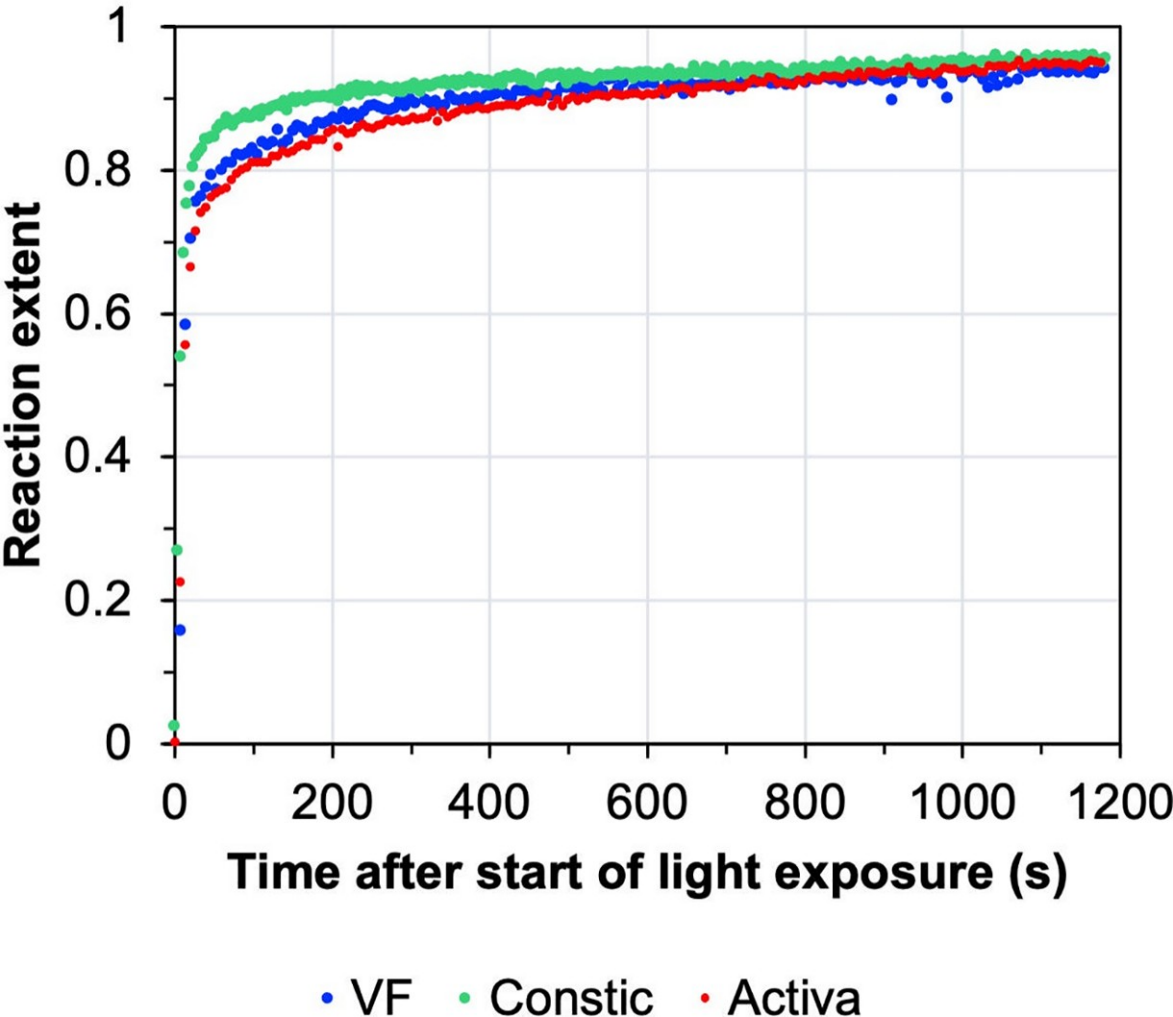
Reaction extent for VF, Constic and Activa. Graph indicates how far the reaction has gone towards its final value at each given time point. Reaction extent reaches 95% of the final value (determined by extrapolation of data versus inverse time to zero) by 1200 s for all the materials.

Regarding kinetics, reaction extent graphs over time, after the start of the light exposure are shown in Fig 4. Results show conversion surpasses 50% of the final value even before light irradiation ends (at 20 s), reaching values higher than 80% after 100 s.

## Discussion

In methacrylates, polymerisation occurs with an opening of a carbon-carbon double bond, leaving a free valence available for reaction with other monomers, which may then co-polymerise. Absorbance changes include shifts in the 1700 cm^-1^ carbonyl region, loss of the 1635–1640 cm^-1^ [*v*(C = C)] peak, and loss of the 1300–1320 cm^-1^ [*v*(C-O)] doublet, with consequent shift to lower wavenumbers [13].

Using the 1635–1640 cm^-1^ peak to monitor this reaction is overshadowed by limiting factors. Firstly, O-H bending vibrations absorb at this wavenumber, invalidating the determination of D_C_ (%) in formulations which contain water [13, 15]. Many published studies measuring D_C_ (%) of dental adhesives using this peak exist, and the results should be cautiously interpreted as they are most probably biased [24–27]. Secondly, shifts in adjacent regions such as the carbonyl 1700–1750 cm^-1^ region affect the [*v*(C = C)] peak band maxima [15]. The findings of this present study also suggest the 1320 cm^-1^ peak to be more reproducible and less prone to systematic errors, when compared to the variability associated with the 1635–1640 cm^-1^ peak region. Such results favour the recommendation of this peak for future D_C_ (%) measurements. Furthermore, in general, most studies use the 1635-1640/1610 combination as the reaction peak and internal reference, respectively, in Bis-GMA, but also, erroneously in UDMA mixtures [28]. The 1610 cm^-1^ is an exclusively aromatic peak, not present in UDMA (Fig 1). Other studies have mentioned the use of the 1535 cm^-1^ bending vibration as the internal reference standard for UDMA D_C_ (%) calculations [29]. However, such peak is also subject to changes during polymerisation, as can be seen in the spectra of Activa. The 1320 cm^-1^ reaction peak and the reference baseline peaks at 1336 and 1350 cm^-1^ chosen in this study are valid for all methacrylates. Despite not being used by the majority of the studies in the dental field, this method has been validated by a considerable number of studies [11, 13, 30–33].

Peak selection is important and will affect not only the results within the same material, but also differences between the materials. This was verified when the 1636–1648 cm^-1^ peak differences were used instead of the 1320 cm^-1^ peak differences, as the former failed to detect differences between the materials, which were present with the other two methods. D_C_ (%) differences are expected as the chemistry of the organic phase of the formulations and filler properties, included in each, differs greatly from material to material [28]. Such differences in specific monomer ratios, their reactivity and glass transition temperatures (T_g_), viscosity of the mixture and also filler load impacts polymerisation kinetics, favouring or limiting reactions [34–36].

Considering D_C_ (%), Activa registered the lowest values, significantly different to VF and Constic when the 1320 cm^-1^ reaction peak method was used. Activa is a dual cure system (light and chemically activated polymerisation), and such systems tend to gradually improve their conversion rates to periods which can go up to 24 h [37, 38]. Besides from chemical and light cure, Activa is claimed to have polyacid components of the glass ionomer family, which undergo an acid-base neutralization reaction. This would mean that Activa has in fact three different setting mechanisms [39]. A triple cure system may be advantageous in scenarios where the material has to be placed in bulk and light activation cannot reach the bottom layers of the material [40]. However, having a mixture of dimethacrylates in a polyacid matrix may not have competing monomer reactivity as seen with other systems, thus decreasing conversion. These results are in line with previous investigations that determined the conversion upon light curing of Activa, which was described as poor [41]. When comparing results achieved with VF in previous studies, their final conversion values were inferior to what was found in this study [42, 43]. Differences may be explained by material thickness and peak selection variability, confirmed with this study. As for Constic, this was the first known study to measure its D_C_ (%). These restoratives belong to a newer class of materials coined self-adhesive composites, which are yet to achieve commercial breakthrough, and are currently being studied [44, 45]. As these materials are formulated with an aim to surpass the need of a dental adhesive, they should come in contact with the tooth and achieve fast polymerisation rates with sufficient monomer-polymer conversion. This allows a densely cross-linked network formation which achieves its final properties, being able to withstand stresses resulting from forces applied in the oral cavity, resistance to degradation and prevention of monomer elution which raises toxicity concerns [34, 46]. Materials which aspire biomimetism should limit their toxicity, which, in this case, can be achieved by reducing the free-monomer elution owing to incomplete polymerisation [47, 48].

The extent of the reaction was similar for all the materials tested, although Constic reached higher initial values, which is in accordance with its polymerisation profile, having achieved a higher final degree of conversion when compared to the other materials. Furthermore, reaction extent results prove that a peak base is not required [11].

The difference spectra shown in this study confirm that the changes seen during the time of spectral acquisition are mainly due to the polymerisation reaction of methacrylates, corroborating findings from previous studies [11, 13]. This is a simple and useful method to understand peak shifts during setting kinetics, although it can be used for a variety of different studies such as changes upon reaction of different materials, biological samples before and after treatment, material mixtures or aging processes [49, 50].

As the ATR-FTIR is highly sensitive to change, factors like atmospheric vapours, such as water, or carbon dioxide from breathing, near the sample measurement, are known to induce noise interference effects on the spectra. These will be observed in the 1700–1600 cm^-1^ region, affecting the 1635–1640 cm^-1^ [δ(O-H)] bend, and also around 3300–3500 cm^-1^ [v(O-H)] for water, or at 2400 cm^-1^ [v(O = C = O)] for carbon dioxide [51]. This may be what was seen in the difference spectra of the materials, further strengthening the need to avoid selecting this peak to monitor conversion in methacrylates. When working with ATR-FTIR systems there is increased difficulty in guaranteeing that there are no variations in water content or temperature during the time of study, as it may not be possible to close the lid of instrument while using ATR accessories, and the sample needs to be accessed for light curing. Atmospheric vapours can thus create noise effects. Instrument manufacturers have tried overcoming this problem by reducing background effects due to water, however this is easily achieved by using the 1320 cm^-1^ peak over the 1635–1640 cm^-1^ region.

These results are also considered highly repeatable, when the appropriate reaction peak/reference are chosen, and are in line with past findings using the ATR technique, that found CV% lower than 2 [52]. This confirms that the ATR-FTIR technique is adequate and sensitive to study polymerisation kinetics of dental composites, and larger variations within the same subset are not expected. Methods of composite preparation should guarantee homogeneity for bulk preparation and packaging [53]. Although heterogeneity may occur in composites, and often results from different filler dispersion within the resin phase, which may lead to differences in light scattering [54]. Within sample variations can also be higher when studying material changes over time [19].

## Conclusion

The ATR-FTIR technique to characterise the polymerisation reaction of methacrylates gives reproducible, sound, results when it is used with the correct peak selection. The 1320 cm^-1^ [v(C-O)] gives reproducible results with less systematic errors, in what concerns variability within repetitions, compared to the traditional 1636 cm^-1^ [v(C = C)]. Calculation of parameters such as DC (%) is largely affected by the peak selection, with material differences being evident only with the 1320 cm^-1^ peak. It is therefore possible to conclude that to assess polymerisation in methacrylates, the 1320 cm^-1^ peak is recommended.

## Supporting information

S1 Dataset Raw data: Monomers, dental composites and kinetics. (XLSX)
